# Efficacy and safety of peginterferon alfa-2a + RBV in cHCV/HIV- vs cHCV-infected patients: interim analysis of a multicenter German cohort

**DOI:** 10.1186/1758-2652-13-S4-O32

**Published:** 2010-11-08

**Authors:** A Baumgarten, T Lutz, P Kreckel, E Wellmann, U Alshuth, S Mauss, J Rockstroh

**Affiliations:** 1 Privat praxis Dupke/Carganico/Baumgarten, Berlin, Germany; 2 Infektiologikum Frankfurt, Frankfurt, Germany; 3 Praxis Koeppe/Kreckel, Berlin, Germany; 4 Roche Pharma AG, Grenzach-Wyhlen, Germany; 5 Center for HIV and Hepatogastroenterology, Düsseldorf, Germany; 6 Universitätsklinikum Bonn, Medizinische Klinik Poliklinik 1, Bonn, Germany

## Purpose of the study

Eradicating cHCV is necessary for the subsequent management of patients with HIV and every HIV/HCV co-infected patient should be considered for treatment. We describe differences between cHCV mono-infected and cHCV/HIV co-infected patients in baseline factors and outcome of cHCV-treatment with peginterferon alfa 2a + RBV in the worldwide largest cHCV cohort.

## Methods

Noninterventional prospective multicenter German cohort, started January 2008 and still recruiting. Interim analysis of cHCV patients, stratified for cHCV mono-infection and cHCV/HIV co-infection. The results are based on a cross-sectional analysis of all available data in April 2010.

## Results

**Figure 1:**
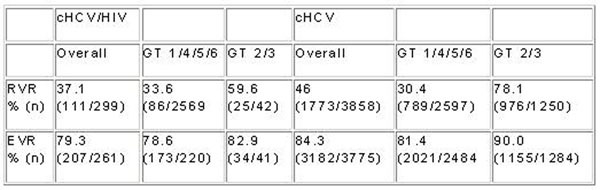


This interim analysis included 5.390 patients, who received HCV-treatment. 397 were cHCV/HIV co-infected (CI) and 4.993 cHCV mono-infected (MI). Main baseline- characteristics: 85.9% were GT1/4/5/6 patients in the CI-Group and 63.4% in the MI-Group, age was 41.0 (CI), 42.0 (MI) yrs, 89.7 (CI), 62.9 (MI)% were male, BMI was 22.8 (CI), 24.9 (MI) kg/m^2^, naïve/relapse/non-responder/re-infection: 86.4/3.8/4.5/5.3(CI), 88.0/6.1/5.3/0.6 (MI) %, source of infection (>1 answer possible): iv drug use 25.2(CI), 44.9(MI) %, sexual transmission 60.7 (CI), 4.1(MI)%, other 8.0 (CI), 24.0 (MI), unknown 13.1(CI), 33.0 (MI)%. 86.4 % of the co-infected patients received antiretroviral HIV-treatment (ART), 66.2 % of them had an HIV-RNA level below 50 copies/mL, median CD4-cells/µ count was 502. From those patients who finished treatment, 52.9% of the CI-Group and 67.7% of the MI-Group completed the planned course. Reasons for discontinuation (>1 answer possible) were non-response (59.3% in CI, 45.5 % in MI) and patient request (24.7% in CI, 14.5% in MI). Other reasons were tolerability (11.1% in CI, 12.0% in MI) and compliance issues (12.3% in CI, 10.5% in MI).Treatment response rates, stratified by genotypes, were already available regarding RVR and EVR (see Figure 1)

## Conclusions

In this preliminary analysis HCV/HIV co-infected patients seemed to respond similar according to RVR and EVR in GT1/4/5/6 as HCV-mono infected patients on HCV-treatment. Treatment discontinuation due to non-response and patient request was much more common in the co-infected group. Other reasons for discontinuation like tolerability and compliance are equal in both arms.A more detailed analysis, in particular the influence of the HIV-ART on HCV-therapy outcome, may help interpreting these data. An updated analysis will be presented.

